# Effects of schooling on aspects of attention in rural Burkina Faso, West Africa

**DOI:** 10.1371/journal.pone.0203436

**Published:** 2018-09-05

**Authors:** Anselme Simeon Sanou, Abdoulaye Hama Diallo, Penny Holding, Victoria Nankabirwa, Ingunn Marie S. Engebretsen, Grace Ndeezi, James K. Tumwine, Nicolas Meda, Thorkild Tylleskar, Esperance Kashala-Abotnes

**Affiliations:** 1 Centre for International Health, Department of Global Public Health and Primary Health Care, Faculty of Medicine, University of Bergen, Bergen, Norway; 2 Department of Public Health, Centre MURAZ Research Institute, Bobo-Dioulasso, Burkina Faso; 3 Department of Public Health, University of Ouagadougou, Ouagadougou, Burkina Faso; 4 Identitèa, Nairobi, Kenya; 5 Department of Epidemiology & Biostatistics, School of Public Health, Makerere University, Kampala, Uganda; 6 Centre for Intervention Science in Maternal and Child Health, Department of Global Public Health and Primary Health Care, Faculty of Medicine, University of Bergen, Bergen, Norway; 7 Department of Paediatrics and Child Health, Makerere University, Kampala, Uganda; University of Wuerzburg, GERMANY

## Abstract

**Background:**

We aimed to study the effects of schooling on aspects of attention using the Test of Variables of Attention (TOVA) among children in rural Burkina Faso.

**Methods:**

We re-enrolled children of a previously community-based cluster randomized exclusive breastfeeding trial in rural Burkina Faso. A total of 534 children (280 boys and 254 girls) aged 6 to 8 years were assessed using the TOVA. We examined the effect size difference using Cohen’s d, ANOVA and conducted regression analyses.

**Results:**

Forty nine percent of the children were in school. Children not in school performed poorly with a small effect size difference for ‘Response Time’, ‘Errors of omission’, and ‘Errors of commission’ compared to children in school. The effect size difference was moderate for ‘Response Time Variability’, and ‘D prime score’.

**Conclusion:**

Schooling affects different aspects of attention in rural Burkina Faso. In settings where literacy and schooling rate is low, public sensitizations of the benefits of schooling need to be reinforced and advice on sending children to school need to be provided continuously.

## Introduction

Attending school is important in child development and is associated with health and increased earnings of offspring [1,2]. It has strong impact on health, life chances, survival, development and children who do not complete school or repeat grades are at the greatest risk [3,4].

However, 59 million school age children do not receive formal education worldwide [5,6] and sub-Saharan Africa has the lowest rate [7]. In Burkina Faso, the net attendance ratio of primary school participation is 50% for female and the enrolment ratio of pre-primary school participation is 4% [7].

Tracking the neurodevelopment of children such as attention irrespective of their exposure to formal education is complicated by the strong association between schooling and performance on neuropsychological measures; several studies using tests administered by human examiner show that neuro-developmental outcomes of children attending school is improved compared to unexposed [8–13].

This paper stemmed from the PROMISE Saving Brains (SB) study, which was a follow-up study of the PROMISE EBF cohorts in Uganda and Burkina Faso [14]. The primary objective of the PROMISE SB study was to assess the long-term effect of exclusive breastfeeding promotion by peer counsellors in Uganda and Burkina Faso, on cognitive abilities, emotion-behaviour-social symptoms, school performance and linear growth among 5–8 years old children. The study showed only small and not significant differences in the outcomes and concluded that peer promotion for exclusive breastfeeding in Burkina Faso and Uganda was not associated with differences in cognitive abilities, emotion-behaviour-social symptoms, school performance and linear growth when children reach school age [15].

Based on the data collected in the PROMISE SB study in Burkina Faso, we explored the effects of schooling on attention in settings where literacy and school attendance is low. To measure attention, we used the Test of Variables of Attention (TOVA) which has similarities with the d2 Sustained-Attention Test [16]. The TOVA is a computerized test measuring attention, that has been used to explore multiple health and developmental risks in the exploration of attention [17–23]. In Africa, the TOVA was used to study attention deficit among children with early cerebral malaria in Senegal [24], and HIV infected children in Uganda [25,26].

## Materials and methods

### Study area, setting, study design and participants

Burkina Faso is a West African low income country. The population aged 0–14 years is 46.3% and 70.1% resides mainly in rural areas [27,28]. The literacy rate is among the lowest in the world [29].

In 2006, a community-based cohort of children was established through The PROMISE Exclusive Breastfeeding (EBF) study in rural Burkina Faso [14,30–32]. The sampling was described [14]. From 2013 to 2015, a study was conducted through the PROMISE Saving Brains study to assess the neuro-cognitive performance of the children from the original cohort who had attained 6–8 years of age; the children from the initial PROMISE EBF trial who were found to be alive and still resident in the study area were re-enrolled as described in detail previously [33].

### Outcome measures

The visual Test of Variables of Attention (TOVA) is an individually administered computerized continuous performance test developed to assess attention in normal and clinical populations. To measure attention in our study, we used the following variables:

- **Response time** (in milliseconds): this score is the measure of the average time it takes for the subject to respond correctly to a target. It is considered as a measure of speed of responding and the reactivity of the subject. A lower ‘Response time’ equates with a faster speed of responding and a swifter reactivity of the subject.
- **Response time variability**: this score is a measure of the variability in the subject’s response time on accurate responses; it is considered as a measure of consistency in the speed of responding. The lower ‘Response time variability’ the more consistent is the performance of the subject.
- **Errors of omission**: this score is measured as the failure to respond to the target stimulus. ‘Errors of omission’ scores are considered to be a measure of inattention. Fewer ‘Errors of omission’ equates with less observed inattention in the subject.
- **Errors of commission**: this score is measured as an inappropriate response to the non-target stimulus. ‘Errors of commission’ scores are considered to be a measure of impulsivity. The higher the ‘Errors of commission’ the more impulsive is the subject’s behaviour.
- **D prime score**: this score is a response sensitivity score and is interpreted as a measure of accurate performance over time. The higher the ‘D prime score’ the greater is the accuracy over time of the subject [25,34–36].

A summary of the calculation’s methods and the scores’ description is presented in Table 1.

**Table 1:** Calculation methods and score description in the TOVA test.

Score	Calculation methods	Calculation formula*	Description
Total response time	Average of the correct response times	$\frac{\sum \left(Correct\;Response\;Times\right)}{\#\;Correct\;Responses}$	Measure of speed of responding and the reactivity
Total response time variability	Standard deviation of the mean correct response times	$\sqrt{\frac{(\sum_{i=0\;}^{n}{\left(x_{i}-Mean\;Correct\;RT\right)}^{2})}{\;(\#\;Correct\;Responses)}}$	Measure of consistency in the speed of responding
Total errors of omission	Number of correctly responds to the stimuli	$\frac{\#\;Omissions}{\left(\#\;Targets-\#\;Anticipatories\right)}x100$	Measure of inattention
Total errors of commission	Number of incorrectly responds to the non stimuli	$\frac{\#\;Commissions}{\left(\#\;NonTargets-\#\;Anticipatories\right)}x100$	Measure of impulsivity
D prime score	Accuracy of stimuli and non stimuli discrimination	$z(\frac{Commission\;Percentage}{100})-z(1-(\frac{omission\;Percentage}{100}))$	Accurate performance over time

*All the calculations are done by the computer and the results are directly given

The test was normed on children and adults, ages 4 to 80+ years and all norms are differentiated by age and gender [33]. The test duration is 22 minutes and the total test time (T) is divided in 4 quarters: quarter 1 (Q1), quarter 2 (Q2), quarter 3 (Q3), and quarter 4 (Q4) and 2 halves, half 1 or H1 where target stimuli are less frequent, and half 2 or H2 where target stimuli are more frequent. The total score reflects subject’s performance over the entire test. Each target stimulus is presented for 100 ms every 2 seconds. In total, 324 target stimuli are presented during the entire test. The target is presented in 22.5% (n = 72) during the first half of the test (stimulus infrequent condition 1) and 77.5% (n = 252) during the second half (stimulus frequent condition 2) [33]. The present study used the TOVA Version 8.1. It was presented on Hp Probook 4540s laptop computers in which Windows 8 was installed. These laptops have 15.6 inches screens for a clear view of the stimuli.

**Fig 1:**
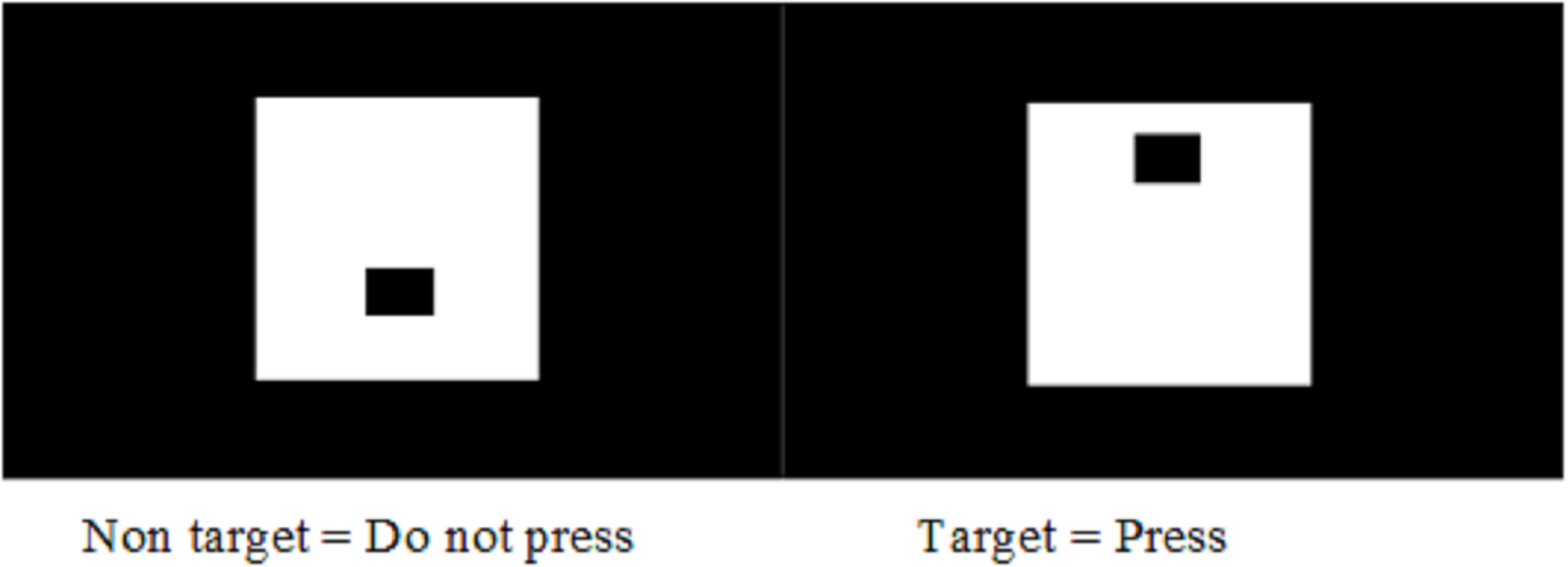
Indication of non target and target in visual TOVA.

The TOVA was individually administered by a team of four psychologists. The instructions were translated in the main local language (Dioula) commonly spoken in the study area. Independent back translations were completed prior to administration to check clarity and veracity. The children were randomly assigned to the different administrators for assessment. Children sat in a quiet room at roughly 75 cm away from the laptop. They were instructed to respond by pressing a hand-held micro switch whenever the target stimulus appears, and not to respond when the non-target stimulus is shown on the screen (Fig 1) [34].

Before starting the TOVA test, a practice which lasts 3 minutes was conducted. Instructions were given until the child understood and passed the practice test. The test-retest reliability of the TOVA is satisfactory after 90 minutes and highly stable after one week [33]. Children were retested on a different day when the test was interrupted.

### Exposure measure

Information about schooling (child attends school yes/no) was collected in a household interview with the caretaker in the same week and prior of the neuro-cognitive assessment. Data collectors approached each child’s household to administer a questionnaire to the child’s caregiver during a one-to-one interview. Mothers were the primary respondents, and responses were verified at the school. Of the 534 children included in this survey, 263 (49.3%) were not in school.

### Covariates

In the interview, questions were asked about additional background characteristics that may influence the child’s performance. These included the child’s age, child’s access to play materials in the home, whether children had been exposed to corporal punishment in the last 12 months, the employment/occupation of the child’s father and of the mother (dichotomized to unemployed = no revenue/ farmer, or employed), the education of the father and of the mother (dichotomized to educated = at least one year in school, or not educated), mother’s age, mother’s depression status using the Hopkins symptom checklist [37] (dichotomized to depression = at least a symptom in the checklist, no depression = no symptom in any of the checklists), and presence of electricity in the compound. Questions regarding past hospitalizations since birth of the child, history of cerebral malaria, were also asked and anthropometric data (height, age) were measured according to standard procedures [38] by a paediatrician at the study site. Stunting was defined as below -2 standard deviations of height-for-age. Information on breastfeeding practices was retrieved from the records of the PROMISE EBF trial.

Field-testing and piloting of all the instruments was conducted prior to data collection to calibrate and standardize the assessment of cognitive measures and the data collection. The psychologists underwent field training and refresher training to standardize the administration of the TOVA on local children prior to the study participants.

### Statistical analysis

Statistical analyses were conducted in several stages using methodologies that were described in detail previously [33]:

1. To examine within population variance, the distribution of scores (mean, standard deviation, median, minimum, maximum, skewness and kurtosis) were used. Covariates differences by schooling were tested using chi square analyses. Box-and-whisker plots were used to illustrate the children’s errors on the TOVA. Extreme scores were winsorized to discount the influence of outliers by replacing their values with the nearest scores within this range.
2. Pearson product-moment coefficients (r) were computed to examine the intercorrelation between the test and the reliability as reported in the TOVA manual through the assessment of the degree of agreement among various test portions, appropriate for measuring reliability for timed tasks such as the TOVA [34].
3. The association between child’s schooling and TOVA attention measures were examined through ANOVA, linear regression and effect size differences (Cohen’s d) [39,40]. A bivariate analysis between potential confounders including age, sex, stunting, past hospitalization, corporal punishment, fathers' education and mothers' employment [8,10,12,41] and the promotion of exclusive breastfeeding (‘intervention arm’ of the initial trial) and the outcome was conducted. All statistical tests were two-sided and declared significant at the 5% level. STATA 13 was used to perform the analysis.

### Ethical considerations

The PROMISE SB study was approved by the Institutional Review Board of Centre MURAZ, BP 390 Bobo-Dioulasso, Burkina Faso number 008-2013/CE-CM on 4^th^ April 2013. Written informed consent was obtained from all caretakers in the study and oral assent was obtained from the children.

## Results

### Study population

**Fig 2:**
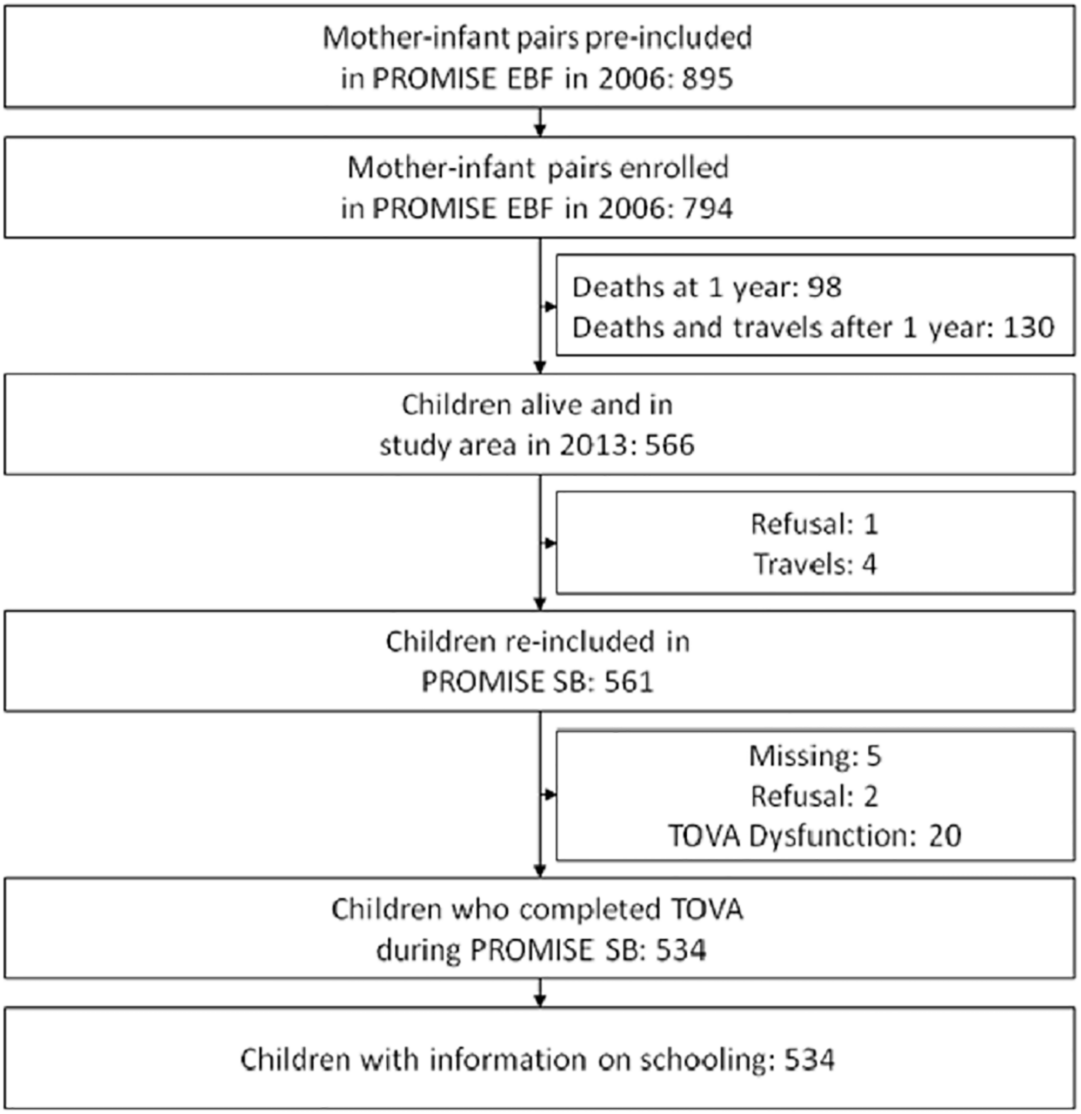
Study profile of children who completed the TOVA and having information on schooling at the PROMISE Saving Brains study in rural Burkina Faso.

As described in detail previously [33], of the original 794 children enrolled in the PROMISE EBF study in Burkina Faso, 561 were found alive and re-consented for the follow-up study; 534 children completed the TOVA and had information on their schooling status (Fig 2).

Of these, 50.7% (271/534) were at school and 52.4% (280/534) were boys. The mean (±*SD*) age at assessment was 7.2 (±0.4 years), the median (IQR) was 7.2 (6.9–7.4) years, with a range of 6.3 to 8 years. The mean (±*SD*) age of the mothers at assessment was 33.4 (±6.3 years) and none of them was educated. Of the fathers, 30.5% (151/495) had attended school and 13.2% (66/500) were in employment. Three quarters of the compounds reported having electricity 77.2%, (386/500) (Table 2). The majority of them had solar power and were not connected to the grid.

**Table 2:** Description of the children who completed the TOVA from the PROMISE Saving Brains study in rural Burkina Faso.

	Total, N = 534 N (%)	Child in school 271 (50.7) N (%)	Child not in school 263 (49.3) N (%)	P value
Age Mean ± *SD* (in years)	7.2±0.4	7.2±0.3	7.2±0.4	0.15
Mothers age Mean ± *SD* (in years)	33.4 ± 6.3	33.3 ± 6.4	33.5 ± 6.2	0.71
Promotion of Exclusive Breastfeeding				0.01
No	283 (53.0)	130 (48.0)	153 (58.2)	
Yes	251 (47.0)	141 (52.0)	110 (41.8)	
Sex				0.08
Boys	280 (52.4)	132 (48.7)	148 (56.3)	
Girls	254 (47.6)	139 (51.3)	115 (43.7)	
Stunting (< -2 *SD* in height-for-age)				0.005
No	435 (84.8)	232 (89.2)	203 (80.2)	
Yes	78 (15.2)	28 (10.8)	50 (19.8)	
Child has been hospitalized				0.008
No	395 (77.2)	188 (72.3)	207 (82.1)	
Yes	117 (22.8)	72 (27.7)	45 (17.9)	
Child has history of cerebral malaria				0.55
No	435 (91.6)	218 (90.8)	217 (92.3)	
Yes	40 (8.4)	22 (9.2)	18 (7.7)	
Child plays with object at home				0.32
No	263 (52.6)	127 (50.4)	136 (54.8)	
Yes	237 (47.4)	125 (49.6)	112 (45.2)	
Child received corporal punishment in the last 12months				0.49
No	477 (95.4)	242 (96.0)	235 (94.8)	
Yes	23 (4.6)	10 (4.0)	13 (5.2)	
Father employed				0.02
Yes	66 (13.2)	42 (16.7)	24 (9.7)	
No	434 (86.8)	210 (83.3)	224 (90.3)	
Father educated				0.99
Yes	151 (30.5)	76 (30.5)	75 (30.5)	
No	344 (69.5)	173 (69.5)	171 (69.5)	
Mother employed				0.004
Yes	26 (5.2)	6 (2.4)	20 (8.1)	
No	474 (94.8)	246 (97.6)	228 (91.9)	
Mothers current depression				0.07
No	261 (53.2)	140 (57.1)	121 (49.2)	
Yes	230 (46.8)	105 (42.9)	125 (50.8)	
Electricity in compound				0.33
Yes	386 (77.2)	190 (75.4)	196 (79.0)	
No	114 (22.8)	62 (24.6)	52 (21.0)	

*SD*: Standard deviation

On the TOVA, the total mean score was 725.5 ± 130.7 for ‘Response time’, 257.9 ± 57.7 for ‘Response time variability’, 78.0 ± 57.5 for ‘Errors of omission’, 27.5 ± 16.5 for ‘Errors of commission’, and 2.3 ± 0.6 for ‘D prime score’ (Table 3). The range was 443.6–1102.4 for ‘Response Time’, 121.3–432.0 for ‘Response time variability’, 1–254 for Errors of omission’, 0–73 for ‘Errors of commission’, and 0.5–4.1 for ‘D prime score’. Within each condition, the data indicated a moderate (r ranges from 0.5 to 0.79) to high reliability (*r*≥0.8) between quarters, halves and with the entire test (*p*<0.001) with a slightly higher correlation coefficient in half2. The intercorrelations reliability coefficients between TOVA test measures were significant between all the tests except Response time—Errors of commission (r = 0.0322) in condition 1 and Response time variability—Errors of commission (*r* = 0.0377) in condition 2 (Table 4).

**Table 3:** Population parameters of performance scores in the d2 Sustained-Attention Test.

	Total, N = 534				Child in school, N = 271				Child not in school, N = 263			
	*M*	*SD*	Skewness	Kurtosis	*M*	*SD*	Skewness	Kurtosis	*M*	*SD*	Skewness	Kurtosis
Total response time	725.5	130.7	0.0002	0.12	701.3	125.7	0.0002	0.58	750.3	131.3	0.09	0.04
Total response time variability	257.9	57.7	0.004	0.98	242.5	50.8	0.32	0.20	273.9	60.1	0.11	0.80
Total errors of omission	78.0	57.6	0.0001	0.80	68.5	52.4	0.0001	0.83	87.8	61.0	0.0001	0.70
Total errors of commission	27.5	16.5	0.0001	0.51	24.8	14.9	0.0001	0.19	30.1	17.6	0.0001	0.69
D prime score	2.3	0.6	0.02	0.83	2.5	0.6	0.04	0.90	2.1	0.6	0.2	0.3

**Table 4:** Intercorrelations reliability coefficients between TOVA test measures as reported by children from the PROMISE Saving Brains study in Cascades health district, rural Burkina Faso.

TOVA test conditions	Condition 1				
Condition 2	Response time	Response time variability	Errors of omission	Errors of commission	D prime score
Response time	1	0.64***	0.46***	0.03	-0.27***
Response time variability	0.68***	1	0.44***	0.36***	-0.47***
Errors of omission	0.44***	0.41***	1	0.13**	-0.63***
Errors of commission	-0.38***	0.04	-0.49***	1	-0.52***
D prime score	-0.14**	-0.45***	-051***	-0.38***	1

**<0.01

***<0.001

### Effects of schooling and covariates on attention measures

Children who were not in school were 49.3% (263/534). Children who were not in school performed more poorly on measures of ‘Response Time’ (mean difference = 49.0 ± 11.1, *p*<0.0001), ‘Response Time Variability’ (mean difference = 31.4 ± 4.8, *p*˂0.0001), ‘Errors of omission’ (mean difference = 19.3 ± 4.9, *p* = 0.0001), ‘Errors of commission’ (mean difference = 5.2 ± 1.4, *p* = 0.0002) and ‘D prime score’ (mean difference = 0.3 ± 0.05, *p*<0.0001) compared to children in school (Table 5). The effect size was small for ‘Response Time’ (Cohen’s *d* = 0.38), ‘Errors of omission’ (Cohen’s *d* = 0.33), and ‘Errors of commission’ (Cohen’s *d* = 0.32). It was moderate for ‘Response Time Variability’ (Cohen’s *d* = 0.56), and ‘D prime score’ (Cohen’s *d* = 0.51) (Table 5). Several covariates including age, sex, stunting, hospitalization and fathers’ education were associated with different aspects of attention measures (Table 6).

**Table 5:** Effect size and bivariate analysis between schooling and TOVA measures of children from the PROMISE Saving Brains study in rural Burkina Faso.

	*df*	*F*	*P*	Mean difference	Cohen *d*	95% CI (*d*)	Bivariate analysis Crude coefficient (95% CI)	
Total response time	1,532	19.45	<0.0001	49.0 ± 11.1	0.38^§^	[0.21–0.55]	49.1	(27.2–70.9)
Total response time variability	1,532	42.81	<0.0001	31.4 ± 4.8	0.56^§§^	[0.39–0.73]	31.4	(22.0–40.9)
Total errors of omission	1,532	15.40	0.0001	19.3 ± 4.9	0.33^§^	[0.16–0.51]	19.3	(9.6–28.9)
Total errors of commission	1,532	13.84	0.0002	5.2 ± 1.4	0.32^§^	[0.15–0.49]	5.3	(2.5–8.1)
D prime score	1,532	35.88	<0.0001	0.3 ± 0.05	0.51^§§^	[0.34–0.69]	-0.3	(-0.4–-0.2)

^§^ Small effect size from 0.2 to 0.49.

^§§^ Moderate effect size from 0.5 to 0.79.

**Table 6:** Crude coefficient between covariates and the TOVA among children from the PROMISE Saving Brains study in rural Burkina Faso.

	Response time	Response time variability	Errors of omission	Errors of commission	D prime score
Age, N	534	534	534	534	534
Crude	-26.3	-13.9	-15.2	-3.9	0.3
95% CI	-57.8–5.3	-27.9–-0.02	-29.1–-1.3	-7.9–0.02	0.1–0.4
p-value	0.1	0.05	0.03	0.05	˂0.0001
Sex, N	534	534	534	534	534
Crude	-26.7	11.0	7.8	1.9	-0.1
95% CI	-48.8–-4.5	1.3–20.8	-1.9–17.6	-0.9–4.7	-0.2 –-0.01
p-value	0.01	0.02	0.1	0.1	0.03
Stunting, N	513	513	513	513	513
Crude	0.4	-8.2	-3.7	5.3	0.2
95% CI	-31.3–32.3	-22.0–5.5	-17.7–10.3	1.4–9.3	0.02–0.3
p-value	0.9	0.2	0.6	0.008	0.02
Hospitalization, N	512	512	512	512	512
Crude	25.3	-13.9	-10.5	2.3	0.09
95% CI	-52.4–1.8	-25.7–-2.2	-22.5–1.4	-1.1–5.7	-0.04–0.2
p-value	0.06	0.02	0.08	0.1	0.1
Corporal punishment, N	500	500	500	500	500
Crude	40.7	-3.2	2.8	-6.1	0.06
95% CI	-14.8–96.2	-27.5–21.1	-21.5–27.2	-13.0–0.7	-0.2–0.3
p-value	0.1	0.7	0.8	0.08	0.6
Father educated, N	495	495	495	495	495
Crude	-31.4	-10.5	-12.0	0.01	0.1
95% CI	-56.8–-6.2	-21.6–0.6	-23.1–-0.9	-3.1–3.2	-0.02–0.2
p-value	0.01	0.06	0.03	0.9	0.09
Mother’s employment, N	500	500	500	500	500
Crude	-6.2	6.4	-15.6	1.6	0.1
95% CI	-58.7–46.2	-16.5–29.3	-38.5–7.4	-4.8–8.1	0.1–0.4
p-value	0.8	0.5	0.1	0.6	0.3
Promotion of EBF N	534	534	534	534	534
Crude	0.2	3.4	2.9	0.8	-0.08
95% CI	22.1–22.5	-6.5–13.2	-6.8–12.8	-2.0–3.6	-0.2–0.03
p-value	0.9	0.5	0.5	0.5	0.1
	Response time	Response time variability	Errors of omission	Errors of commission	D prime score
Age, N	534	534	534	534	534
Crude	-26.3	-13.9	-15.2	-3.9	0.3
95% CI	-57.8–5.3	-27.9–-0.02	-29.1–-1.3	-7.9–0.02	0.1–0.4
p-value	0.1	0.05	0.03	0.05	˂0.0001
Sex, N	534	534	534	534	534
Crude	-26.7	11.0	7.8	1.9	-0.1
95% CI	-48.8–-4.5	1.3–20.8	-1.9–17.6	-0.9–4.7	-0.2 –-0.01
p-value	0.01	0.02	0.1	0.1	0.03
Stunting, N	513	513	513	513	513
Crude	0.4	-8.2	-3.7	5.3	0.2
95% CI	-31.3–32.3	-22.0–5.5	-17.7–10.3	1.4–9.3	0.02–0.3
p-value	0.9	0.2	0.6	0.008	0.02
Hospitalization, N	512	512	512	512	512
Crude	25.3	-13.9	-10.5	2.3	0.09
95% CI	-52.4–1.8	-25.7–-2.2	-22.5–1.4	-1.1–5.7	-0.04–0.2
p-value	0.06	0.02	0.08	0.1	0.1
Corporal punishment, N	500	500	500	500	500
Crude	40.7	-3.2	2.8	-6.1	0.06
95% CI	-14.8–96.2	-27.5–21.1	-21.5–27.2	-13.0–0.7	-0.2–0.3
p-value	0.1	0.7	0.8	0.08	0.6
Father educated, N	495	495	495	495	495
Crude	-31.4	-10.5	-12.0	0.01	0.1
95% CI	-56.8–-6.2	-21.6–0.6	-23.1–-0.9	-3.1–3.2	-0.02–0.2
p-value	0.01	0.06	0.03	0.9	0.09
Mother’s employment, N	500	500	500	500	500
Crude	-6.2	6.4	-15.6	1.6	0.1
95% CI	-58.7–46.2	-16.5–29.3	-38.5–7.4	-4.8–8.1	0.1–0.4
p-value	0.8	0.5	0.1	0.6	0.3
Promotion of EBF N	534	534	534	534	534
Crude	0.2	3.4	2.9	0.8	-0.08
95% CI	22.1–22.5	-6.5–13.2	-6.8–12.8	-2.0–3.6	-0.2–0.03
p-value	0.9	0.5	0.5	0.5	0.1

## Discussion

In the present study, we observed an association between children being in school and better attention as measured by the ‘Response time’, the ‘Response time variability’, the ‘Errors of omission’, the ‘Errors of commission’ and the ‘D prime score’ of the TOVA computerized neuropsychological performance test among children aged 6 to 8 years in rural Burkina Faso compared to children in school.

Our study was conducted in an African context where it is not uncommon for school age children to not be in school. All the children were from the general population in rural areas in Burkina Faso and were previously part of a community-based cluster randomized trial which assessed the promotion of exclusive breastfeeding [14]. These results were supported by the evidence of sensitivity to within population variance and robust reliability of the TOVA in our context. In its first application in the country, we found variation in performances in the TOVA measures. Children were positively engaged in carrying out the test.

Concerning test reliability, the comparison of scores on test sections quarters, halves for both stimulus infrequent and frequent condition with the total scores was highly comparable to the data reported in the TOVA manual [34]. The reliability coefficient on half 2 was slightly higher relative to the reliability coefficient on half 1. This might be explained by the practice effects obtained from completing the first half, as also found in other studies [17,20]. Also, the correlation coefficients indicate that some of the TOVA measures are not sufficiently reliable, which is particularly true for the Response time variability score. This is consistent with other research; For example, a study found that the performance variability measures in the d2 attention test should be interpreted with caution as they lack reliability [16]. Another study demonstrated (by means of simulation analysis on grounds of classical test theory) that measures of performance variability can never achieve the same degree of reliability as compared to measures of central tendency (i.e., mean scores) [42].

In and not-in school children had the same mean age. The differences found between schooled and unschooled children is consistent with the effects of schooling seen in the performance on non-computerized neuropsychological measures [10,11,13].

The mechanisms in the literature potentially underlying the effects of schooling on attention measures can be divided into three categories: the global effects, the specific effects and the test-taker effects. The aspect of (1) global effect on attention abilities is the measurement intention of psychometric test such as TOVA and is based on instructional experience [43,44]. As soon as children start school, they are required to sit still in order to make progress with learning the cultural techniques (reading, writing, arithmetic), they learn to focus their concentration on relevant aspects for a certain period of time; They learn to concentrate and to resist distractions in terms of a general ability. Studies showed that school attendance measured more finely by additional days in school have been associated with increase scores of intelligence tests [8,10–13,41,45,46]. School exposure has also been associated with other beneficial effects on brain development [10] and yields important development benefits and improves health, earning, human capital [47–50]. However, the interpretations are post hoc and cannot be validated in the study.

The aspect of (2) specific effects is based on the constant and repeated exercise of these cultural techniques which lead to the development of specific skills; this might also contribute to the observed performance differences between the groups and is not entirely avoidable.

The aspect of (3) test-taker effects is based on the understanding of what is being demanded of them. Studies show that exposure of children in school to the process of receiving and using instructions for learning and education improves test performance by increasing the understanding of the test taker of what is being demanded of them [51,52]. In our study, the instructions were given by trained and experienced psychologists. We consider this as a strength of our study as recent research has pointed on the importance to verbally explain task requirement and to instruct the participants to give their best possible performance, in contrast to written instructions. By this means, the experimenter is able to obtain immediate feedback from the participant, and if necessary, can deliver further explanation to ensure that they have understood the instruction correctly [53]. The assessment in our study was based on a standardized computerized measure of attention for children which has been used in Africa [24–26]. In fact, studies suggest that computerized neuropsychological performance tests provide many advantages over tests administered by a human examiner. Observed increases in reliability and validity [54,55] stem from a reduction in human error [56], increased ease of administration [57], less time devoted to the preparation of the materials, reduction in errors during scoring [58], increased accessibility for specific populations [59], ability to measure performance on time-sensitive tasks, and automated data exporting [56,60].

The study has some limitations. The participants were part of an established community-based cohort of children as described in detail previously [14,30–33]. Given the non-random selection of schooled and un-schooled children in the general population, selection bias should not be omitted. In our study, we experienced equipment malfunction mainly due to power shortages, with a difficulty to reschedule the children for another TOVA assessment; the missing values on TOVA were, however, random. A specific limitation of the uses of TOVA in similar contexts is related to the need for special equipment, a secured area for testing, which requires a constant electricity supply. Another limitation is the lack of information on the overall validity of the measure which was used for the first time in our context.

We still consider this paper to be important as it highlights the need to raise awareness of the benefits of schooling in rural contexts without implying a causal link between schooling and cognitive performance. Due to the large number of factors simultaneously affecting cognitive performance in children in Africa, we cannot completely isolate the true effect of schooling from other influences as there are a multitude of potential covariates that naturally cannot be controlled in the present study. However, in the context of schooling, teachers and educators may have an important role in advising the public on its potential benefits. Sensitization initiatives need to be reinforced and advice on sending children to school need to be provided continuously. This study also continues to highlight the need to address educational experience in analyzing and interpreting child neuropsychological performance indicators. Those working in areas where compulsory education exists and is well followed may fail to take into account the consistent effect that schooling has on test performance. Hence, this study might be considered a valuable contribution to our knowledge as it addresses severely neglected aspect which deserves serious attention in the future.

## Conclusion

Schooling affects different aspects of attention in rural Burkina Faso. In settings where literacy and schooling rate is low, public sensitizations of the benefits of schooling need to be reinforced and advice on sending children to school need to be provided continuously.

## Supporting information

S1 File Schooling and attention measure dataset. (ZIP)
